# Study Protocol: The Behaviour and Pain in Dementia Study (BePAID)

**DOI:** 10.1186/1471-2318-11-61

**Published:** 2011-10-17

**Authors:** Sharon Scott, Louise Jones, Martin R Blanchard, Elizabeth L Sampson

**Affiliations:** 1 Marie Curie Palliative Care Research Unit, UCL Mental Health Sciences Unit University College Medical School, 1st Floor, Charles Bell House, 67-73 Riding House Street, London, W1W 7EJ, UK; 2 Barnet Enfield and Haringey Mental Health Trust, St Ann's Hospital, St Ann's Road, London, N15 3TF, UK; 3 UCL Mental Health Sciences Unit, University College Medical School, 1st Floor, Charles Bell House, 67-73 Riding House Street, London, W1W 7EJ, UK

## Abstract

**Background:**

People with dementia admitted to the acute hospital often receive poor quality care particularly with regards to management of behavioural and psychiatric symptoms of dementia (BPSD) and of pain. There have been no UK studies on the prevalence and type of pain or BPSD in people with dementia in this setting, or on how these may impact on patients, carers, staff and costs of care.

**Methods/Design:**

We shall recruit older people with dementia who have unplanned acute medical admissions and measure the prevalence of BPSD using the Behave-AD (Behaviour in Alzheimer's Disease) and the CMAI (Cohen Mansfield Agitation Inventory). Pain prevalence and severity will be assessed by the PAINAD (Pain Assessment in Advanced Dementia) and the FACES pain scale. We will then analyse how these impact on a variety of outcomes and test the hypothesis that poor management of pain is associated with worsening of BPSD.

**Discussion:**

By demonstrating the costs of BPSD to individuals with dementia and the health service this study will provide important evidence to drive improvements in care. We can then develop effective training for acute hospital staff and alternative treatment strategies for BPSD in this setting.

## Background

Dementia is common in older people admitted to acute hospitals in the United Kingdom (UK), affecting 42% of adults over 65 years with an unplanned medical admission. These patients have high mortality with a quarter of those with severe impairment dying during the index hospital admission [1]. Dementia significantly increases the length of hospital admission [2-5], complications [4] and the risk of iatrogenic harm through polypharmacy [6]. A number of recent documents including the English National Dementia Strategy, the National Dementia Research Summit and Alzheimer's Society "Counting the Cost" report have raised concerns regarding the quality of care received by people with dementia in acute hospitals and have highlighted lack of original research in this field [7-9].

### Behavioural and psychological symptoms of dementia in the acute hospital

The term "behavioural and psychological symptoms of dementia" (BPSD) encompasses a range of symptoms including agitation, aggression, delusions, hallucinations, depression and apathy. These are common in dementia, multifactorial in origin and often secondary to complex interactions between the severity of dementia, the environment and other illness [10]. BPSD are extremely distressing for the patient and difficult to manage in the busy acute hospital. They may lead to the inappropriate use of antipsychotic drugs, increasing the risk of stroke, falls and death [11].

Carers have given rich reports on how BPSD may worsen during hospital admission[9]. However, although there is some qualitative research, [12] in our recent systematic review [13], we found no studies on the type, severity or frequency of BPSD in the acute hospital, how hospital staff manage these symptoms and the impact on patients. These data are vital if we are to develop and evaluate effective non-pharmacological interventions for BPSD in the acute hospital.

### Pain

Pain is commonly under detected and undertreated in people with dementia [14,15]. Many clinical staff believe that people with dementia actually experience less pain [16]. This may occur because people with dementia are unable to express clearly that they are in pain. Under-treatment of pain may lead to protective responses such as aggression, distress and agitation, vocalisations or depression and withdrawal [17]. It may increase the risk of delirium [18], slowing recovery and increasing functional decline [16]. In acute hospitals in the UK it is not usual clinical practice to assess routinely whether people with dementia are in pain. However, this may be a worthwhile approach as when pain assessment scales are used in dementia patients the use of analgesics increases significantly [19].

### The relationship between BPSD and pain

Behavioural problems in people with dementia may be an expression of unmet needs such as boredom, fear, discomfort or pain [20]. However, the relationship between BPSD and pain is poorly understood. The perception and communication of pain is a complex process and particular behaviours are not exclusively associated with pain. In people with dementia such behaviours may also indicate embarrassment, depression or distress. "Pain behaviours" therefore lack specificity and some "pain scales" may actually be detecting broader distress. To understand any direction of causality, it is important to use self-report and observational/behavioural pain scales concurrently [14]. More work is required to establish whether the use of pain tools is feasible in the acute hospital, whether these tools are reliable in detecting pain and whether there is a relationship between pain, particularly that which is undetected and undertreated, and BPSD.

## Methods/Design

### Aims

Our aim is to examine the impact of behavioural and psychological symptoms (BPSD) and pain, during an acute hospital admission, in people with dementia. We shall explore two specific areas. First, how behavioural and psychological symptoms (BPSD) affect outcomes for the person with dementia, informal carers and the hospital, and second the detection and management of pain in people with dementia.

### Objectives

Our main objectives are to examine, in older people with dementia admitted to acute hospital wards:

• the prevalence and types of BPSD

• how hospital staff respond to and manage these symptoms

• the impact of BPSD on costs of care

• the impact of BPSD on the person with dementia, for example the prescription of antipsychotic drugs, the length of hospital stay, quality of care and the risk of adverse events

• prevalence of pain and how well this is detected and managed by hospital staff

• the time spent by informal carers assisting nursing staff with basic care tasks

Finally, we shall test the hypothesis that there is an association between BPSD and pain, particularly whether BPSD are a manifestation of under-detected or under-treated pain.

## Methods

This protocol describes an observational cohort study.

### Setting

To ensure our population is representative we will recruit from two London hospitals. Both cover a large area of London encompassing socioeconomic and ethnic diversity, serving a population of two million people, six primary care trusts and four mental health trusts. The hospitals have differing strengths and weaknesses in terms of their Care Quality Commission ratings and are at different stages of implementing the English National Dementia Strategy with varying provision of liaison psychiatry.

#### Sample size

We were unable to find studies in this setting on which to base our power calculation. We therefore used data from a community study of pain in people with dementia that used the Cohen Mansfield Agitation Inventory (CMAI) [21]. To analyse the hypothesised association between pain and BPSD we will use repeated measures (every 4 ± 1 days). Power depends on the correlation between measurements which we are unable to predict. We calculated a conservative estimate by considering a perfect correlation between repeated measurements (ρ = 1). Shega et al. [22] reported a mean CMAI score of 50.5 and 42.5 in patients with and without pain respectively (standard deviation 18.9). Assuming the presence of pain in 55% of our sample [21], the power to detect a significant difference with 250 patients would be 91%. To ensure an adequate sample size for our other analyses we have taken a point prevalence of BPSD of 31% from a community based sample of people with dementia [10]. We will need to recruit 250 patients (125 from each hospital) to ensure a 95% confidence interval for prevalence estimates of BPSD with an acceptable 6% precision.

### Identification of participants

**Figure 1:**
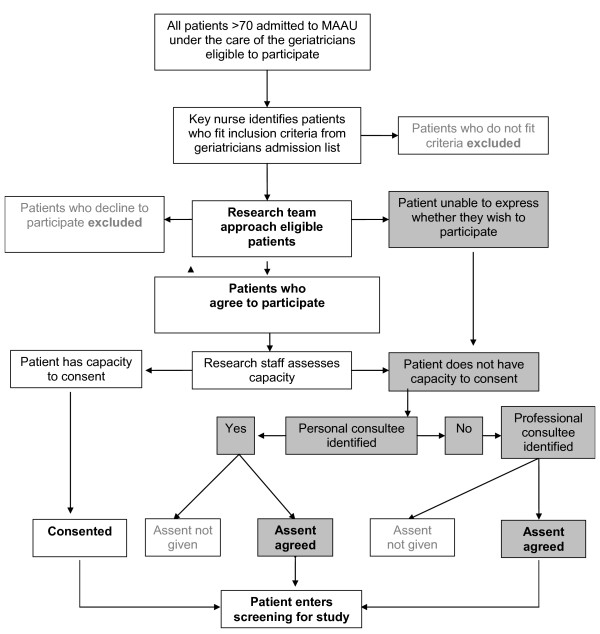
**Participant recruitment and consent procedure**.

In both hospitals, all patients are admitted to the medical acute admissions unit (MAAU), before transfer to the care of the elderly wards. Two research assistants will spend four months at each site and see all patients admitted to each MAAU who are under the care of the geriatricians within 72 hours of admission. The geriatricians will provide a list of people who have been admitted under their care over the previous 24 hours. Research assistants will ask ward nursing staff to identify patients from this list who fit the inclusion criteria, see Figure 1.

### Inclusion criteria

• patients over the age of 70 who have an unplanned acute medical admission

• able to give verbal consent or an informal carer or "professional consultee" available to give assent

• sufficient English language to complete the study ratings

• Abbreviated Mental Test Score (AMTS [23]) of ≤ 7/10 (routinely measured on admission)

### Exclusion criteria

• patients who indicate either verbally or non-verbally that they do not wish to participate

• patients consistently rated as being delirious during the study without a previous diagnosis of dementia

• patients who are moribund, in a coma, non-English speaking (the assessment tools selected for this study have not been translated or validated in languages other than English) or where there are clinical concerns that may preclude them being approached

#### Screening

All potential participants will be screened for delirium using the Confusion Assessment Method (CAM) [24], (Table 1). Those who are not delirious will be consented to the study and have a MMSE assessment. If their score is ≤ 24 they will be entered into the study. Patients with delirium will be screened again 48 hours later, if this has resolved they will undergo testing with the MMSE. If they remain persistently delirious they will not be eligible to participate as we will be unable to establish whether or not they have an underlying dementia. Patients with delirium but who have a clearly documented previous diagnosis of dementia in the hospital notes will enter the study.

**Table 1:** Study Assessment Tools

**Confusion Assessment Measure (CAM) **[24]	Has a sensitivity of over 94% and specificity over 90% for detecting delirium. Distinguishes accurately between delirium and dementia.
**Abbreviated Mental Test Score (AMTS) **[23]	Global cognitive assessment tool recommended for screening patients admitted to hospital. Maximum score of 10 cut off ≤7.
**Mini-Mental State Examination (MMSE) **[33]	Most widely used screening test for cognitive impairment. Maximum score of 30 cut off ≤24.
**Functional Assessment Staging Scale (FAST) **[25]	Describes a continuum of 7 successive stages of functional impairment, from normality to the most severe dementia.
**Charlson Co-Morbidity Index (CCI)**[34]	Calculates severity of chronic co-morbidity. Includes 19 diseases weighted on the basis of their association with mortality, allowing for the documentation of painful co-morbidities ^43^
**Behave-AD **[26]	Covers 7 domains of BPSD including delusions, hallucinations, affective disturbance and aggressiveness. Includes a global rating of the trouble these behaviours cause to caregivers.
**Cohen Mansfield Agitation Inventory (CMAI) **[27]	This rates a range of behaviours many of which are relevant and challenging on hospital wards, for example wandering, grabbing on to people and pushing. It enables measurements over short timescales.
**Pain Assessment in Advanced Dementia (PAINAD) **[28]	Validated observational pain tool that measures pain during care tasks and at rest.
**Faces Pain Scale **[29]	Self report pain scale consisting of line drawings of 6 faces indicating increasing amounts of pain. Can be used by people with advanced dementia.
**Assessing Care of Vulnerable Elders (ACOVE) **[31]	Validated indicators of the quality of hospital care received by vulnerable older people. Designed to be gathered from hospital notes post patient discharge.

If we were to exclude dementia patients with delirium we would risk excluding those patients who are most likely to have the greatest symptom burden and the study would not be truly representative of patients admitted to acute hospital wards in the UK.

#### Consent

Many patients will be acutely ill. They will have a dementia, delirium or both and will not be able to give fully informed consent. Therefore our consent procedure has been developed to comply with capacity legislation governing England and Wales (Mental Capacity Act 2005). Our study was approved by the ethics committee of Central London REC3 which is "flagged" to consider research on those unable to consent for themselves. This is an observational study and the risk of harm to the patient is negligible.

1. If a patient agrees to participate we will conduct a brief, structured assessment of their capacity to consent. If they have capacity to consent we will obtain written informed consent from them.

2. If they do not have capacity to consent we will attempt to identify their next of kin, carer or someone close to the person to give proxy assent. If we cannot contact a next of kin within 48 hours of initial screening we will contact a professional consultee for assent (professional consultees will be defined as a senior member of the clinical care team who is not directly involved in the research or patient's care).

#### Study procedures

### Study measures after consent

Dementia diagnosis will be confirmed using the operationalised DSM-1V criteria and dementia severity measured using the Functional Assessment Staging Scale (FAST) [25]. The reason for admission, co-morbidities, medication (cholinesterase inhibitors, analgesia and neuroleptics) and demographics (i.e. place of residence) will be obtained from medical notes. Patients will undergo observational assessment for BPSD (Behave-AD and Cohen-Mansfield Agitation Inventory-CMAI[26,27]). Pain will be assessed using a combination of methods concurrently. The Pain Assessment in Advanced Dementia (PAINAD) observational behavioural pain scale [28] will be used to assess pain at rest and during a care task activity, followed by the self-reporting assessments, the question "Do you have pain at this moment"? and the Faces pain scale [29] (see Table 2). Pain and BPSD measures will be obtained independently by separate researchers.

**Table 2:** Study Assessment Schedule

Measures	Source	Baseline	Follow-up 4 ± 1 days	Discharge	Death
Confusion Assessment Method (CAM)	1,2,3,4	*			

Abbreviated mental test score (AMTS)	3	*			

Mini mental state examination (MMSE)	1	*			

DSM1V dementia criteria	1,2,3,4	*			

Functional Assessment Staging Scale (FAST)	1,2,3,4	*			

Charlson co-morbidity index	4	*			

Demographics	4	*			

Reason for admission	4	*			

Place of residence	4	*		*	

Waterlow score	4	*		*	

Pressure ulcers & grade	4	*		*	*

Use of parenteral feeding	4	*		*	*

Continence	4	*		*	*

Behave-AD	1,2,3,4	*	*		

Cohen Mansfield Agitation Inventory (CMAI)	1,2,3,4	*	*		

BPSD precipitants	1,2,3,4	*	*	*	*

BPSD Non-pharmacological management	3,4	*	*	*	*

BPSD medication	4	*	*	*	*

Dementia medication	4	*	*	*	*

Pain Assessment in Advanced Dementia (PAINAD)	1	*	*		

"Are you in pain" yes/no pain question	1	*	*		

Faces pain scale	1	*	*		

Possible precipitants for pain	1,2,3,4	*	*	*	*

Analgesics prescribed	4	*	*	*	*

Adverse events	4			*	*

Length of admission	4			*	*

Assessing Care of Vulnerable Elders (ACOVE)	4			*	*

Carers questionnaire	2			*	*

Economic Data	4			*	*

1-Patient, 2-carer if present, 3-health professionals, 4-patient care records

BPSD- behavioural and psychological symptoms of dementia

#### Subsequent study assessments

Patients will be reviewed every 4 ( ± 1 days) and the assessment for BPSD and pain repeated. Notes will be reviewed and discussions held with the patient's key nurse to identify any possible cause for pain, e.g. constipation or injury, adverse incidents, the context and precipitants for any BPSD e.g. during personal care tasks and how staff dealt with any BPSD (for example with sedatives) or managed pain (i.e. analgesia prescribed).

We will calculate the inter-rater reliability for the pain and BPSD tools at regular intervals during the study.

### On discharge from hospital

Data will be collected from notes and will include adverse events, measured using validated pre-defined criteria[30], falls, length of stay, whether antipsychotic or pain medication was prescribed on discharge, and whether there was a change of residence after discharge. The ACOVE (Assessing Care of Vulnerable Elders) tool will be used to assess quality of care. This examines 16 specific quality indicators in general hospital care and geriatric-prevalent conditions (e.g., dementia, delirium, pressure ulcers) and allows the calculation of adherence rates for care processes (screening, diagnosis and treatment) [31].

### Carer involvement

When the person with dementia has been discharged from hospital, we will send a brief questionnaire to the identified carer or next of kin asking questions about their experiences of visiting their relative whilst in hospital. We will clearly state that this will be anonymised and will not be directly fed back to the clinical team. We will not collect any personal data on the carers. The ethics committee agreed that we will not ask them to give signed consent; we will assume that if they have completed the questionnaire and return it to us, they have consented to participate.

#### General principles of data analysis

We shall use simple descriptive statistics for the demographic features of the cohort using Chi squared, t-tests or non-parametric statistics if indicated. For prevalence estimates of BPSD we will conduct a sensitivity analysis, excluding patients whose BPSD appeared only in the context of delirium. For some analyses we will create overall "indicator" variables i.e. one to denote presence of any BPSD at any time during the admission. Outcomes will be divided into clinically relevant sub groupings i.e. falls, use of medications (antipsychotics, sedatives) or prolonged length of stay above the median. We shall use logistic regression to analyse the relationship between our principle exposures, i.e. BPSD and outcomes such as falls, controlling for potential confounders identified through clinical experience and bivariate analyses. Appropriate corrections will be made for multiple comparisons. For the analysis of the overall relationship between pain and BPSD we will use Generalised estimating equations (GEE) as we are using multiple observations on the same individual and analyses are clustered by participant. GEE is advantageous for use in the context of this project as it does not require a "balanced" design i.e. observations on all occasions on each individual.

#### Health economics

We shall compare the average cost of care per hospital admission for patients with and without BPSD. The analysis of hospital costs will include all costs incurred during the admission, such as hotel costs, nursing, specialist consultations, investigations, surgery, and additional agency staff.

Unit costs will be obtained from the hospital finance department, and applied to estimates of resource use. These may vary in between the hospitals, which may result in different average costs per admission.

#### Staff training

We recruited two research associates, ensuring that they had experience of working with people with dementia and in the busy acute hospital environment. They were trained by ES and SS over a period of two weeks. This included in-depth discussion and practice on all of the study assessment tools, data collection and the process of assessing mental capacity and obtaining consent for people who do not have capacity, approaching carers and the use of professional consultees according to the MCA (2005).

#### Pilot study

The pilot study took place over a period of two weeks and assessed the following: the identification and screening of patients, the consent process (including procedures for informal carers and professional consultees to give assent), practice and use of selected assessment tools and the collection of data from varying sources. This demonstrated that the study recruitment schedule was both practical and feasible and that we were able to, on average, recruit eight participants per week (the number required to achieve an adequate sample size).

## Discussion

When older people with dementia are admitted to hospital with acute medical illness, it can be distressing for them and their families. Symptoms such as pain may be poorly detected and managed and often BPSD such as agitation and aggression are exacerbated in this busy environment. In turn disturbed behaviour in people with dementia may be problematic for other patients and health professionals. BPSD and pain may result in, and be the result of, poor quality of care. There may also be economic costs to the hospital. The need to conduct research in this field has recently been highlighted by a number of government reports and strategies [32]. However, conducting clinical studies of people with dementia in the acute hospital poses considerable challenges to researchers and this may explain why we found little previous evidence of work in this setting [13].

### Challenges within the acute hospital environment

The acute hospital is an extremely busy environment in which to conduct research, with patients being rapidly assessed, transferred to MAAU and then moved to care of the elderly wards, sometimes all within hours of admission. This makes it difficult to track study participants and obtain accurate reports of their condition; staff may not have had time to fully assess the patient's BPSD and continuity and consistency of information is affected by staff shift changes. We have overcome many of these challenges by building good relationships with doctors, nurses and allied health professionals on the MAAU and care of the elderly wards. Before starting, each unit and ward was provided with study information folders and researchers met and discussed the study with staff, answering questions or concerns. Researchers work closely with ward clerks to identify where patients have been transferred or discharged to.

### Challenges related to capacity and consent

The development of this study gave us the opportunity to explore ways in which we could recruit patients with dementia who, by the nature of their illness, may not have the mental capacity to give informed consent. From the literature and personal communication with other researchers in the field we were aware that gaining ethical approval for similar studies has proved challenging. We therefore explored and negotiated various ways of obtaining consent (or proxy assent) within ethical guidelines whilst balancing the need to be able to do research in this very difficult area. We followed advice from the Mental Capacity Act (2005) Code of Practice i.e. assessing the person with dementia using a structured approach for testing capacity, seeking assent from a carer when the person had lost capacity and using professional consultees when a carer could not be contacted. The pilot study demonstrated that this method of obtaining consent was both practical and feasible within the acute setting. Our methods may prove useful to other researchers embarking on similar projects in the future.

### Challenges in supporting study staff

There is a high volume and rapid turnover of patients, and research workers screen numerous patients to identify potential participants. This involves, the assessment of capacity, contacting carers or professional consultees to give assent within a short window of opportunity (72 hours of admission). The researchers may have a large number of follow-up assessments to complete on a daily basis.

In addition researchers may occasionally witness poor quality care, for example, when pain or BPSD are not managed appropriately by staff, and not be able to directly and immediately intervene. This is not an intervention study and there is the potential for a Hawthorne effect around study participants. However, procedures have been put in place for the reporting of perceived sub-standard clinical care to the study supervisors who then follow a standardised procedure to inform the appropriate senior clinical staff. Supportive regular clinical supervision is provided within the research team.

### Importance of support from other agencies

The success of this study depends upon support from senior staff in NHS trusts. This work may be perceived as potentially critical to the clinical teams involved, but they were willing for us to research the clinical care delivered in their organisations. The Alzheimer's Society Quality Research Monitors (mainly former family carers of people with dementia) are provided by our funding organisation to help ensure that our study gains a perspective from people with dementia and their carers. Their advice has been vital in developing our proxy assent procedures and they continue to actively participate in our study steering group meetings.

## Final conclusions

We have overcome numerous challenges in the development of this study, particularly in regard to consent procedures and the large number of potential study participants who need to be screened. The study will help to fill in many of the gaps we have identified in the current literature regarding the effects of BPSD and pain during acute hospital admission on older people with dementia. Improved detection and treatment of pain with adequate analgesia may be an effective strategy for treating BPSD, without necessarily resorting to antipsychotic medications.

A better understanding of these symptoms, their impact and costs will drive more effective treatment strategies and investment in services, to improve the standard of hospital care and the quality of life for people with dementia.

## Competing interests

The authors declare that they have no competing interests.

## Authors' contributions

ELS, LJ and MB devised the project and obtained funding. ELS, SS, LJ and MB wrote the study protocol. SS managed the piloting of the study and with LJ and ELS refined the study processes. All authors read and approved the final manuscript

## Author information

Sharon Scott MSc, RGN, Senior Research Nurse

Louise Jones FRCP Head of Marie Curie Palliative Care Research Unit

Martin R Blanchard MD MRCPsych Senior Lecturer in Old Age Psychiatry

Elizabeth L Sampson MD MRCPsych, Clinical Senior Lecturer

## Pre-publication history

The pre-publication history for this paper can be accessed here:

http://www.biomedcentral.com/1471-2318/11/61/prepub
